# Polymerization-Induced Phase Segregation and Self-Assembly of Siloxane Additives to Provide Thermoset Coatings with a Defined Surface Topology and Biocidal and Self-Cleaning Properties

**DOI:** 10.3390/nano9111610

**Published:** 2019-11-13

**Authors:** Jaleh Mansouri, Vi Khanh Truong, Shane MacLaughlin, David E. Mainwaring, Graeme Moad, Ian J. Dagley, Elena P. Ivanova, Russell J. Crawford, Vicki Chen

**Affiliations:** 1 UNESCO Centre for Membrane Science and Technology, School of Chemical Engineering, University of New South Wales, Sydney, NSW 2052, Australia; v.chen@uq.edu.au; 2 Cooperative Research Centre for Polymers, Notting Hill, VIC 3168, Australia; vi.khanh.truong@rmit.edu.au (V.K.T.); dagley@bigpond.net.au (I.J.D.); 3 Faculty of Science, Engineering and Technology, Swinburne University of Technology, PO Box 218, Hawthorn, VIC 3122, Australia; demainwaring@swin.edu.au (D.E.M.); elena.ivanova@rmit.edu.au (E.P.I.); 4 Nanobiotechnology Laboratory, School of Science, College of Science, Engineering and Health, RMIT University, Melbourne, VIC 3001, Australia; russell.crawford@rmit.edu.au; 5 BlueScope Steel Research, Port Kembla, NSW 2505, Australia; Shane.Maclaughlin@bluescopesteel.com; 6 CSIRO Manufacturing, Clayton, VIC 3168, Australia; 7 Defence Science and Technology, Department of Defence, 506 Lorimer Street, Port Melbourne, VIC 3207, Australia; 8 School of Chemical Engineering, University of Queensland, Brisbane, QLD 4072, Australia

**Keywords:** polyester coatings, siloxane copolymer additives, cleanability, antibacterial, surface segregation, dynamic nano-topography

## Abstract

In this work, we report on the incorporation of a siloxane copolymer additive, poly((2-phenylethyl) methylsiloxane)-co(1-phenylethyl) methylsiloxane)-co-dimethylsiloxane), which is fully soluble at room temperature, in a rapid-cure thermoset polyester coating formulation. The additive undergoes polymerization-induced phase segregation (PIPS) to self-assemble on the coating surface as discrete discoid nanofeatures during the resin cure process. Moreover, the copolymer facilitates surface co-segregation of titanium dioxide pigment microparticulate present in the coating. Depending on the composition, the coatings can display persistent superhydrophobicity and self-cleaning properties and, surprisingly, the titanium dioxide pigmented coatings that include the siloxane copolymer additive display high levels of antibacterial performance against Gram-positive (*Staphylococcus aureus)* and Gram-negative (*Pseudomonas aeruginosa*) bacteria. This antibacterial performance is believed to be associated with the unique surface topology of these coatings, which comprise stimuli-responsive discoid nanofeatures. This paper provides details of the surface morphology of the coatings and how these relates to the antimicrobial properties of the coating.

## 1. Introduction

Antimicrobial polymeric materials include materials that are inherently antimicrobial, materials that are chemically modified by surface treatment with polymers/monomers that confer antimicrobial activity, and polymers that are blended with organic or inorganic biocidal/antiadhesion additives [1,2]. The surface treatment approach, which is more environmentally friendly, typically involves the application of functional polymer coatings and brushes [3]. This approach is mainly used for the development of anti-adhesion surfaces that reduce the degree of bacterial attachment to the surface. On the other hand, polymers with quaternary ammonium groups (cationic biocidal moieties) are probably the most explored class of polymeric biocides [4]. Polysiloxanes are well known for their efficiency in reducing the formation of biofilms via their fouling-release characteristics [5,6,7] as a result of their low surface energy, low modulus and high flexibility [8,9,10]. The incorporation of inorganic slow-release biocides into polysiloxanes has been shown to be effective in reducing the degree of biofouling taking place on surfaces in the coating industry [9,10,11,12].

One of the most effective inorganic antimicrobial additives is titanium dioxide (TiO_2_). It is well-known that the treated (passivated) TiO_2_ absorbs UV radiation and photochemically protects the polymer materials in which it is incorporated [13]. Untreated TiO_2_, is inherently photocatalytic, as it generates free radicals when it is exposed to specific wavelengths of light [14]. Antibacterial photocatalytic coating formulations containing untreated anatase titanium dioxide (TiO_2_) nanoparticles have been studied extensively over the last few decades for a variety of applications [12,14,15,16,17]. The effect of particle size, crystallinity and type of UV light on the level of antibacterial performance have been thoroughly investigated. A more recent approach to render a surface to exhibit antibacterial properties is to introduce a specific nanostructure in a post-treatment process [18,19]. It was shown that the naturally occurring nanoscale high aspect ratio surface features on cicada and dragonfly wing epicuticle lipids exhibited highly bactericidal behavior [20]. Learning from nature, it was proposed that the nano-"bed of nails" surface killed bacteria during the attachment process, where the bacterial cell wall ruptured at a point between the nanopillar features on the wing surface during the attachment process [21,22]. Li introduced an analytic thermodynamic model to explain the bactericidal mechanism of nanopatterned surfaces [23]. The approach involved analyzing the role of the total free energy change taking place as the bacterial cells adhere to the patterned surface. Surfaces possessing a nanopatterned structure exhibit greater levels of bactericidal behavior than planar surfaces because of the increase in contact adhesion areas between the surface and the bacteria. 

In other work, it was demonstrated that controlling the interpillar spacing in fabricated polyethylene terephthalate nanopillar arrays could promote the bacterial adhesion, when the spacing was much smaller than the diameter of bacterial cells, or inhibit the adhesion when the spacing approached the diameter of the cells [24]. Furthermore, nanopillar arrays were found to deform the morphology of bacteria, in particular, the diameter, length, and side curvature, indicating a possible correlation between bacterial adhesion and cell morphology. Generating spatially organized microtopographic surface patterns on a poly dimethyl siloxane (PDMS) coating resulted in lower degrees of bacterial adhesion and biofilm formation in comparison to that obtained on an untreated surface [10,11,25]. It was shown that by changing the surface topography with a lithographic a post-treatment approach, the degree of bacterial adhesion could be reduced by 40%, regardless of the extent of surface wettability. 

The initial purpose of this work was to design copolymer additives that could be incorporated in relatively small amounts of surface coating formulations to impart self-cleaning properties through the development of an appropriate surface topology during the curing stage of the coating. With this aim, a variety of potential copolymer additives were screened. Critical factors were deemed to be the additive solubility in the coating formulation as well as some immiscibility in the cured composition (to facilitate phase segregation and self-assembly into discrete domains). We envisaged that the additive should be of sufficiently low molar mass to come to the surface during a rapid cure process and a sufficiently high molar mass would be retained on the surface to provide permanent surface modification for the coating. Certain additives produced by Evonik attracted our attention for their known use in providing permanent surface modification (scratch resistance) in materials produced by a melt processing process. For example, incorporation of only 2% Tegomer^®^ M-Si 2650 into polycarbonate/acrylonitrile-butadiene-styrene (PC/ABS) blends resulted in the formation of a scratch-resistant surface [26]. This behavior was attributed to processing-induced surface segregation.

The use of siloxane additives to form modified topologically active surfaces have been previously reported [10,11,18,27,28]. To the best of our knowledge, however, this is the first time that such surfaces with a specific nanostructure have been produced by a process of polymerization-induced phase segregation and self-assembly of an additive system in a rapid cure pigmented thermoset coating composition. In this work, we report on the self-assembly of a specific siloxane copolymers additive, which influences the topology, self-cleaning, and antibacterial performance of the coating.

## 2. Materials and Methods 

### 2.1. Polyester Coating Compositions 

The coating formulations used in the study are a proprietary polyester-melamine coil coating comprising polyester polyols, melamine-formaldehyde crosslinkers, crosslinking catalysts, and solvents with or without added pigment. These were formulated according to the principles and methods commonly used in the industry [29]. The non-pigmented polyester-melamine coating is labeled “Control 1” and pigmented polyester-melamine coil coating is labeled “Control 2”. (See the illustration in SI-1).

A passivated (aluminosilicate coated) titanium dioxide microparticle (rutile) structure was used as the white pigment in the preparation of a pigmented polyester-melamine coil coating. The average size of the microparticles was 250 nm, measured from field emission scanning electron microscopy (FESEM) images using image analysis software ImageJ (Figure S1 and Table S1).

### 2.2. Copolymer Additive

Poly((2-phenylethyl) methylsiloxane)-co(1-phenylethyl) methylsiloxane)-co-dimethylsiloxane) (TEGOMER^®^ M-Si 2650 (EVONIK, Essen, Germany) is a statistical copolymer with the ratio of monomer units, determined by ^1^H NMR, as being 1:2.7:34.8. The number average molar mass (*M*_n_) and weight-average molar mass (*M*_w_) for the additive, as determined by gel permeation chromatography (GPC), were 6890 and 10,630 polymethyl methacrylate (PMMA) equivalents, respectively. The modified compositions used in this study were prepared by incorporation of TEGOMER^®^ M-Si 2650 (Essen, Germany) to the Control 1 and Control 2 samples (See SI-1). 

### 2.3. Sample Preparation

The Control 1 and Control 2 samples were prepared by coating the formulations on primed stainless steel or treated aluminum panels using a stainless-steel drawdown bar, which was part of an automatic casting machine (Sheen 1133N, TQC Sheen GmbH, Hilden, Germany) at the drawing speed of 500 mm/s. They were then baked in an oven at a peak metal temperature (PMT) of 230 °C for 30 s. The panels were then quenched in water (80 °C for one min) and dried at room temperature overnight. The dry coating thickness was determined to be between 18–22 µm measured by a high-resolution digital micrometer (Mitutoyo) with an accuracy of ±1 µm. For preparation of the modified coating compositions, TEGOMER^®^ M-Si 2650 was completely dissolved in the polyester-melamine formulation at different weight percentages based on the solids content of the formulation and then coated and cured under the same conditions used for preparing the Control samples. A detailed preparation method is given in the Supporting Information (SI-1 and Figure S2).

### 2.4. Characterization Methods

#### 2.4.1. Surface Microstructure

FESEM observations of the surface were made using a field-emission gun scanning electron microscope (NOVA NanoSEM 450, FEI Company, Hillsboro, OR, USA), at an accelerating voltage of 5 kV and working distance of 5 mm. For the surface morphology observation, a small section of the sample was mounted on carbon tape and sputter-coated with chromium. At least 3 areas for each duplicate sample were investigated. The elemental analysis of the coating was carried out using energy-dispersive x-ray spectroscopy (EDS) analysis feature from NanoSEM 450 fitted with Bruker SDD-EDS detector at accelerating voltage of 15 kV. 

#### 2.4.2. Surface Topography and Nanomechanical Investigation in Air and Fluid Environments

A FastScan Dimension^®^ Icon^®^ atomic force microscope (AFM) (BRUKER, Billerica, MA, USA) with ScanAsyst™ in tapping mode was used to study the surface topography and surface micromechanical properties of the cured coatings in air and liquid. A FastScan-C probe was used. 

The AFM force spectroscopy is a quantitative technique for the characterization of local/surface mechanical properties of the coating. The force-distance curves (with information on tip-sample interaction) can be used to calculate sample mechanical properties such as Young’s modulus, adhesion, and deformation using tip parameters and suitable models of contact mechanics [30]. 

The tip (made from silicon) had a nominal end radius of 5 nm, with a nominal spring constant of 0.8 N/m and a nominal resonance frequency of 300 kHz. The cantilever was made from silicon nitride. Samples were scanned with a peak force tapping rate of 1–2 kHz and an imaging rate of 1–2 Hz. All scanned images were ‘flattened’ using a plane-fit procedure to remove the large-scale waviness from the roughness analysis. The arithmetic mean roughness (Ra), root mean square roughness (R_rms_) and maximum height (R_max_) were calculated using a data processing unit. PeakForce QNM (Quantitative NanoMechanics) mode was used for simultaneous recording of topography and force curves for each pixel by applying Derjaguin–Müller–Toporov (DMT) model [31]. Modulus and adhesion forces were obtained from the relevant channels that reflected real-time forces with each tip-surface contact.

#### 2.4.3. Surface Wetting Properties and Surface Energy 

The change in surface properties of coatings was also examined through changes in the water contact angle. Static water contact angles were measured using a contact angle goniometer (Attension^®^ Theta Lite, BioLin Scientific. Stockholm, Sweden) by the sessile drop method. OneAttension^®^ software was used for data acquisition. Reported values are the average of at least three separate measurements. 

To obtain the different components of the surface energy, polar and non-polar liquids were used. The surface free energy (and the split between different components of the surface free energy) were quantitatively determined from the interactions taking place between the surface and a series of probe liquids of different and known interfacial properties, using Van Oss-Chaudhury-Good theory. Probe liquids for measuring surface energy were distilled water, glycerol and diiodomethane (DIIM) and the typical droplet sizes were 2 μL, 6 μL, and 1.5 μL, respectively. For more detail please refer to the Supporting Information (SI-2 and Table S2)

#### 2.4.4. Surface Chemical Composition

To investigate the surface chemistry of the coatings, an X-ray photoelectron spectroscopic (XPS) analysis was performed using a Thermo ESCALAB250i high-resolution X-ray Photoelectron Spectrometer (ThermoFischer, VG Scientific, Hillsboro, OR, USA) surface analysis system. A photoelectron take-off angle of 90° was used. The X-ray radiation source was monochromated Al Kα, 1486.6 eV, 200 W. Data was recorded in the “Fix Analysis Transmittance” (FAT) mode. For wide scans, pass energy of 100 eV was used, and for region scans, a pass energy of 20 eV was used. C1s at 285.0 eV was used as the binding energy reference. The untreated samples were mounted on copper sample stubs by means of double-sided adhesive tape. Under these conditions, no charging effect was observed for any of the samples. The detection depth of XPS is typically about 5–10 nm [32].

#### 2.4.5. Dirt Resistance 

The dirt resistance of coatings (cleanability) was assessed using the difference in the colour of the coated surface before and after contamination with carbon black slurry. Coatings with the least change were ranked as having ‘excellent dirt resistance’ and those exhibiting maximum changes are ranked as having ‘poor dirt resistance’. The dirt resistance was quantified using a dirt pick-up (DPU) analysis test (also known as carbon slurry test). The method used is described in the Supporting Information (SI-3 and Table S3).

#### 2.4.6. Antimicrobial Properties 

The quantification of the interactions taking place between the topology of the coating surface and the model bacteria was carried out by incubating two bacteria species, *Staphylococcus aureus* CIP 65.8 and *Pseudomonas aeruginosa* ATCC 9721 for 18 h in the presence of these surfaces. The samples with bacteria were incubated at 25 °C and in a “dark” incubator to eliminate any photocatalytic effects. The method is described in more detail in the Supporting Information (SI-4).

## 3. Results and Discussion

### 3.1. Coating Compositions Based on Non-Pigmented Polyester (Control 1)

The modified coating compositions were initially prepared by the incorporation of 5 wt.% TEGOMER^®^ M-Si 2650 into the non-pigmented coating formulation (Control 1), with the objective of investigating the coating surface properties and performance in the absence of the white pigment (titanium dioxide). The curing conditions used were the same as those used for preparing the pigmented coating composition, as described in the Experimental Section.

**Figure 1:**
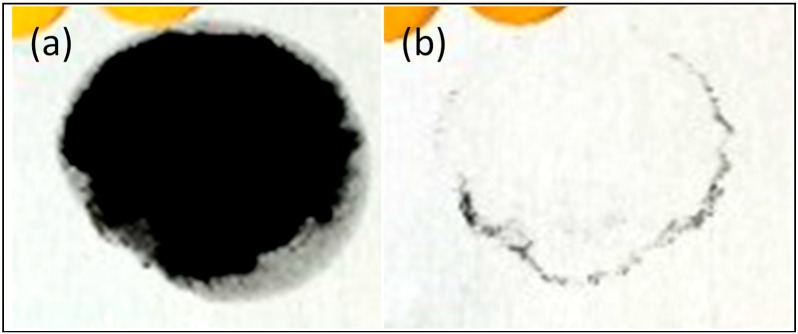
Images of Control 1 with 5 wt.% of siloxane additives after contamination with carbon black (**a**) before and (**b**) after rinsing with water.

Both the non-pigmented coating (Control 1) and the siloxane copolymer modified non-pigmented coating composition exhibited very good dirt resistance, with almost all the contamination (a dry carbon slurry being used as model dirt) being able to be rinsed off under running water. Images of the panels in Figure 1 for the modified coating show a very clean surface after rinsing off the carbon slurry with water. This may be due to the presence of a high concentration of nitrogen on the surface, a result of self-condensation and surface enrichment of melamine. This is evident from XPS analysis for the modified Control 1 sample, which contains a higher concentration of nitrogen in comparison to that of the pigmented coating. The high concentration of melamine was expected to increase the number of glassy areas on the surface, hence reducing the extent of adhesion of the dirt particles [33,34].

**Figure 2:**
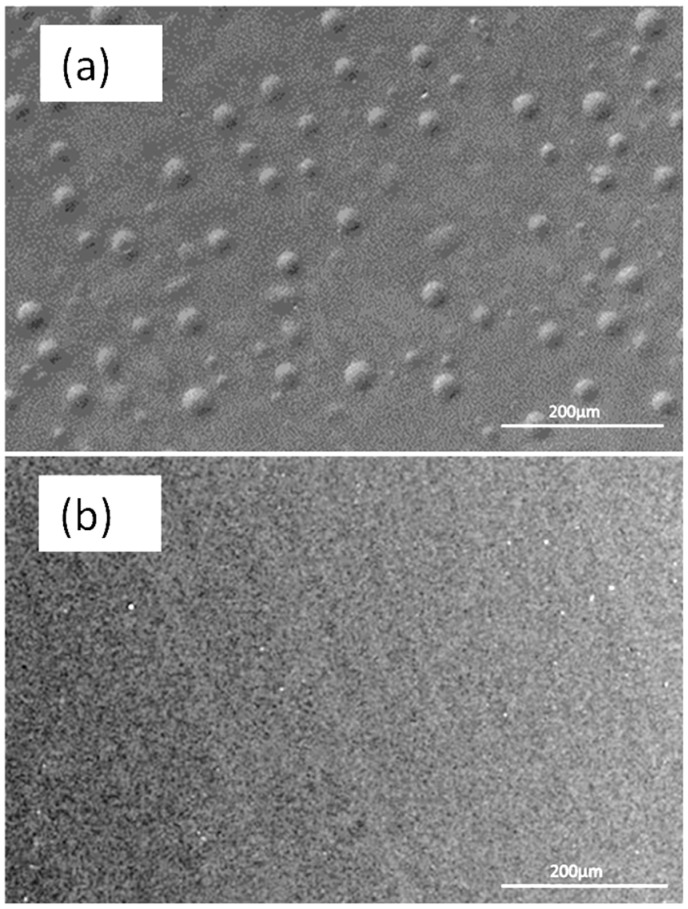
Surface morphology of (**a**) Control 1 coating modified with siloxane additive (TEGOMER^®^ M-Si 2650) and the (**b**) Control 1 coating.

Changes in the surface microstructure and surface wettability of coating were evident after the incorporation of the siloxane copolymer. FESEM images (Figure 2) show the formation of isolated semi-spherical regions on the surface of the coating prepared using the modified coating composition. This may be the result of the self-assembly and migration of the siloxane additive to the surface of the coating. 

The results of contact angle and surface energy analyses are given in Table 1, which shows the significant changes taking place in the wettability of the Control 1 sample after the incorporation of the TEGOMER^®^ MSi 2650 (siloxane additive). The water and glycerol contact angles were increased indicating that the wettability against polar liquids decreased and the contact angle with the non-polar liquid (DIIM) decreased, confirming that the modified coating was less polar at the surface due to migration of siloxane. The surface energy of the modified coating was found to be higher than that of Control 1. The higher surface energy of modified coating in spite of migration of low surface energy siloxane copolymer is the result of the change in microstructure and higher roughness as is presented in Figure 2. 

A surface chemical analysis using XPS showed the presence of an average 6.5 atomic percentage of silicon at the surface of the modified Control 1 coating (with 5 wt.% siloxane additive) confirming the surface segregation of siloxane copolymer. In summary, there were changes in the bacterial cells surface chemistry, surface wettability, and surface microstructure because of the incorporation of the siloxane copolymer to the Control 1 coating. 

**Figure 3:**
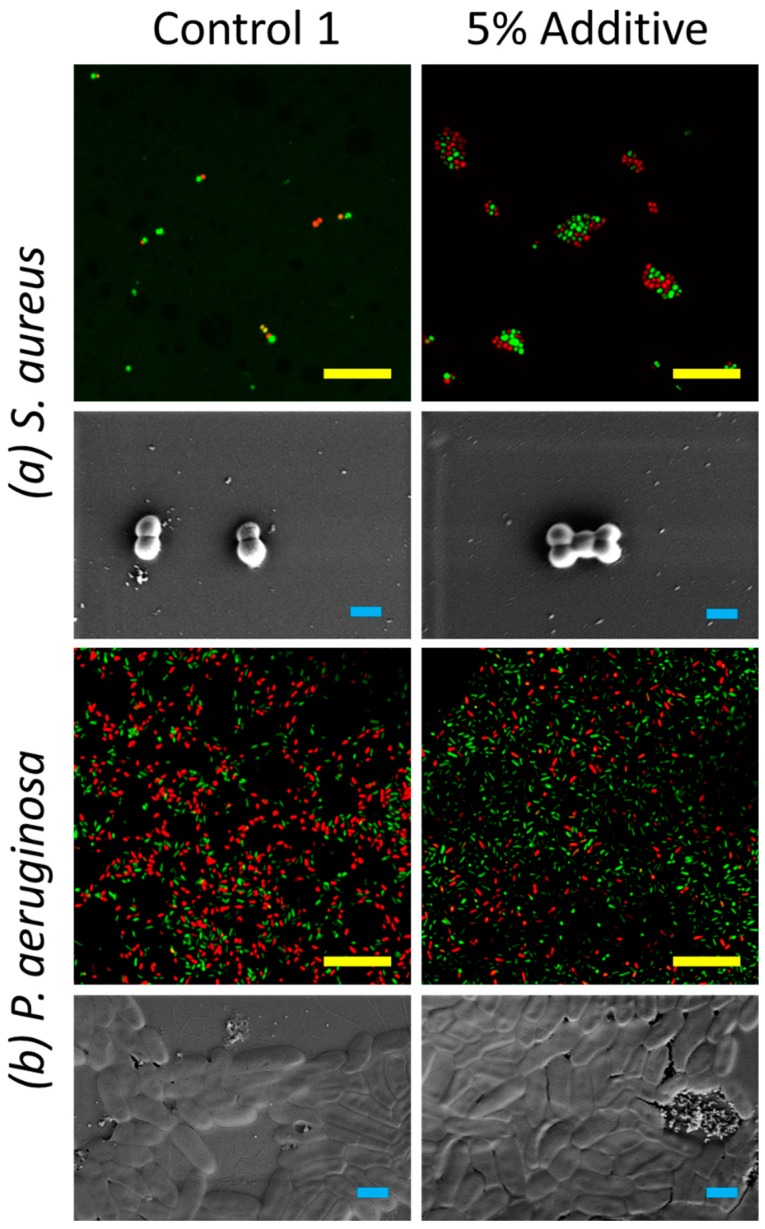
Attachment patterns and viability of (**a**) Gram-positive *S. aureus* and (**b**) Gram-negative *P. aeruginosa* bacterial cells on the Control 1 and Control 1 samples containing the 5% additives. Confocal laser scanning microscopy (CLSM) micrographs (green indicating viable cells, and red indicating non-viable cells) highlighed the viable and non-viable bacterial cells on these surfaces (scale bar 20 µm). Scanning electron micrographs (bottom) display the morphology of bacterial cells on these surfaces (scale bar 1 µm).

Antimicrobial activity studies showed that the incorporation of the siloxane copolymer decreased the biocidal activity and increased the extent of cell attachment taking place on the surface, with the biocidal effect decreasing significantly against *Pseudomonas aeruginosa* bacteria (Figure 3). The extent of bacterial attachment taking place on the surface substantially increased for *Staphylococcus aureus* bacteria, as shown from the data presented in Table 2. The difference in these observations may be due to the thinner layer of cell wall of *P. aeruginosa* in comparison to *S. aureus* bacterial cells [21,22]. In Control 1 with 5% additive, surfaces become more nanoscopically rough, and this may have led to the increase in *S. aureus* attachment. In contrast, the surface did not influence the extent of attachment of the *P. aeruginosa* bacterial cells. It was found that the extent of nanoroughness had an influence on the antibacterial efficiency of the surface, as found in previous studies [19,35,36].

### 3.2. Coating Compositions Based on the Pigmented Polyester (Control 2)

To investigate the effect of the additive on the antibacterial activity of the coatings containing the pigmented formulations, coating compositions were prepared by the addition of siloxane copolymers (TEGOMER^®^ M-Si 2650) to the Control 2 (Section SI-1) formulation at concentrations of 5, 9 and 11 wt.%. 

**Figure 4:**
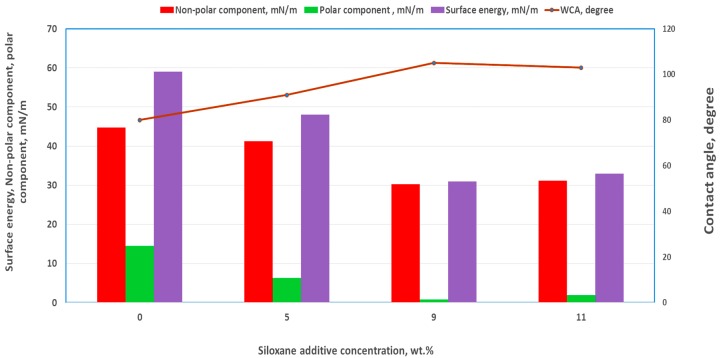
Water contact angle and surface energy components of the Control 2 and Control 2 samples with TEGOMER^®^ M-Si 2650.

The results presented in Table 3 and Figure 4 show that the surface energy of the pigment-coated surfaces was reduced as the concentration of additive was increased. Both the polar and dispersive components of the surface energy were found to have reduced, especially at additive concentrations higher than or equal to 9 wt.%. The contact angle between the non-polar liquid, DIIM, and the modified coated surface increased significantly when high concentrations of additive had been incorporated into the formulation. For the control pigmented coated (Control 2) surface, the contact angle of DIIM was approximately 30°, indicating its non-polar nature, while incorporation of 11 wt.% additive resulted in a low surface polarity, with contact angle of 56° being recorded for DIIM. The results show that coatings containing 5, 9 and 11 wt.% additive displayed higher degrees of surface hydrophobicity compare to that of the Control 2 coating. These changes in the surface properties may suggest that the self-assembly took place on the surface because of the polymerization-induced segregation of the siloxane additive. Self-assembly polymerization-induced phase separation has been reported earlier when the additive became insoluble during polymerization [37].

**Figure 5:**
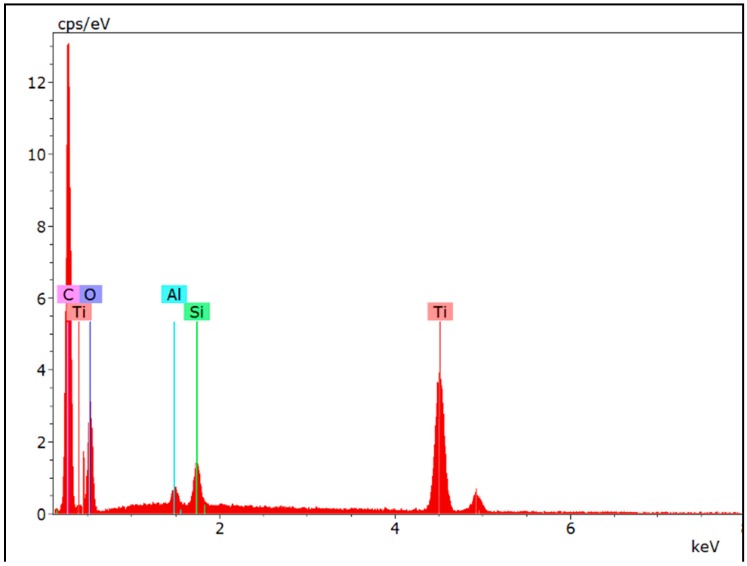
EDS spectra for pigmented coating modified with 5 wt.% siloxane copolymers.

The surface chemical composition for the Control 2 coating and the various coatings prepared using different concentrations of TEGOMER^®^ M-Si 2650, analyzed by XPS, are summarised in Table 4. These results show that the greater the concentration of additive, the greater the amount of silicon (siloxane) that is present on the surface. Furthermore, nitrogen (from polyester-melamine resin) was not detected on the surface for the coating containing the 11 wt.% of siloxane additive, which demonstrates the significant presence of siloxane. The results are consistent with the observed higher water contact angles and the relatively non-polar surface behavior of this coating composition. The surface chemistry results also highlighted that for coatings containing the titanium dioxide pigment, a smaller amount of melamine being present at the surface (e.g. lower atomic percentage of nitrogen). Furthermore, at the same concentration of siloxane additive (5 wt.%), greater amounts of siloxane appeared to migrate to the surface on the coatings containing the pigmented polyester-melamine coating than was observed for the non-pigmented coating. This may suggest a synergistic effect taking place between the passivated titanium dioxide, causing the surface segregation of the siloxane copolymer. EDS analysis of the coating (Figure 5) showed the presence of titanium and aluminum, confirming that the titanium dioxide pigment is present at approximately few microns below the surface of coating. The ratio of Al to Ti is around 8. 

The change in surface chemistry and surface energy has led to better cleanability performance of the modified compositions, examined by a dirt-resistance test based on the procedure outlined in SI-3. DPU results given in Table 5. It shows that incorporation of TEGOMER^®^ M-Si 2650 (at all concentrations), improved the self-cleaning performance of pigmented formulations. DPU for modified coating was less than −20 vs. DPU of −30 for “Control 2” coating.

**Figure 6:**
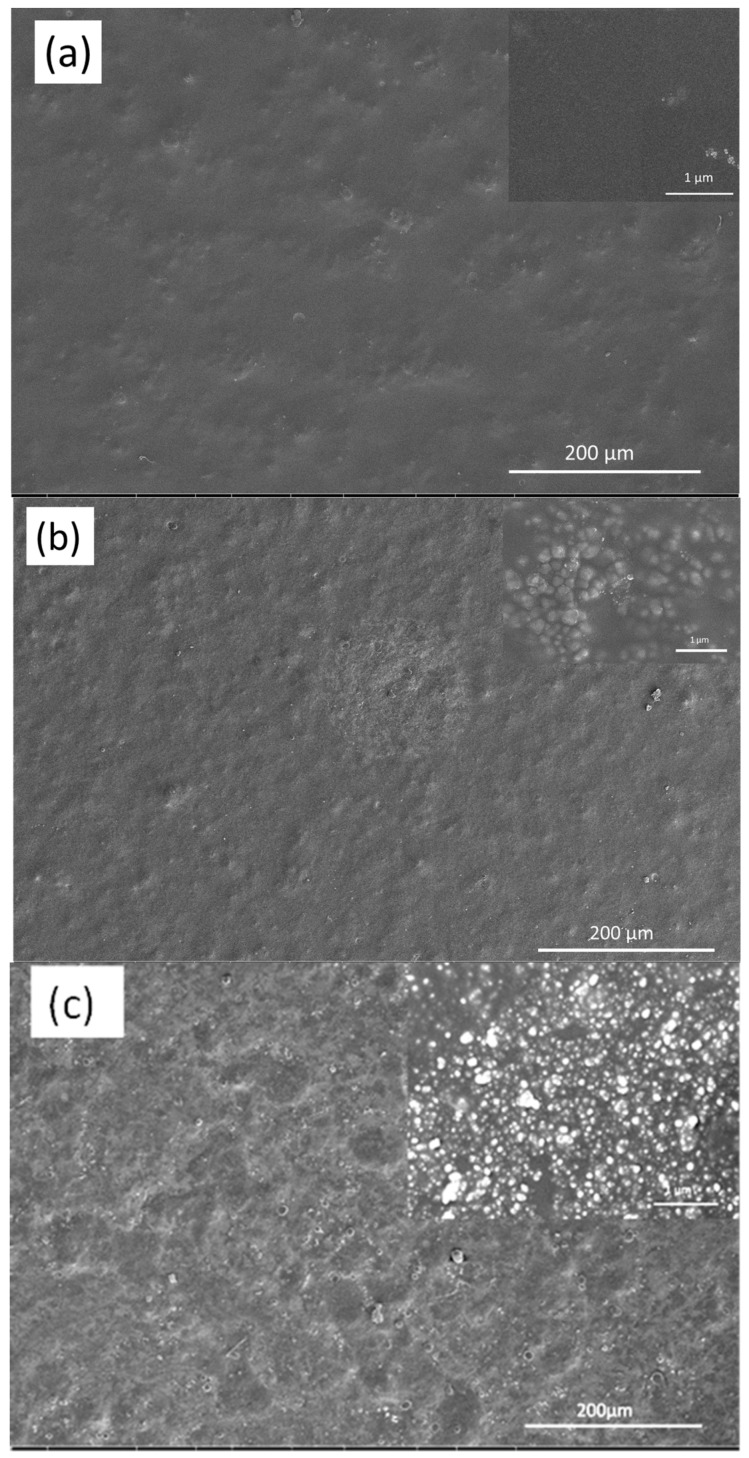
Surface morphology of (**a**) the Control 2 coating, and the Control 2 coating containing (**b**) 5 wt.% and (**c**) 11 wt.% siloxane additive.

In Figure 6, SEM images obtained for the Control 2 coating, shown in Figure 6 highlighted the presence of a relatively smooth and uniform surface, with some valley-like areas present on the surface, with few surface particles being observed. Incorporation TEGOMER^®^ MSi 2650 into the surface coating resulted in a coating that had some microparticles and droplets/agglomerates appearing on the surface. As the concentration of additive was increased from 5 to 11 wt.%, the number of agglomerates/microparticles present on the surface significantly increased. These significant changes may be attributed to the interaction of the siloxane additive with the titanium dioxide nanoparticles and other components of the coating since this structure was not present in the absence of the pigment.

**Figure 7:**
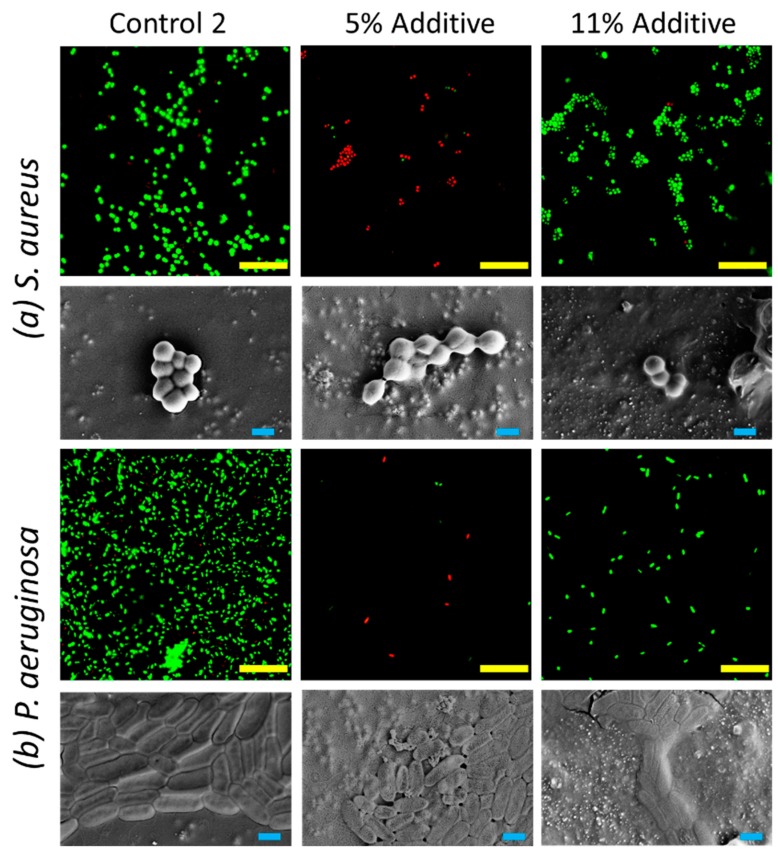
Attachment patterns and viability of (**a**) Gram-positive *S. aureus* and (**b**) Gram-negative *P. aeruginosa* bacterial cells on Control 2 and Control 2 with 5% and 11% additives samples. CLSM micrographs (green indicating viable cells, and red indicating non-viable cells) highlighted the viable and non-viable bacterial cells on these surfaces (scale bar 20 µm). Scanning electron micrographs (bottom) displayed the morphology of bacterial cells on these surfaces (scale bar 1 µm).

It appears that the siloxane additive facilitated the migration of titanium dioxide particles to the surface, causing the formation of polymeric agglomerates during the fast curing stage of the modified polyester coating. This resulted in an increase in surface roughness (Table 6) and a decrease in surface energy as discussed earlier. This change in micro- and nanostructure resulted in a significantly lower degree of *P. aeruginosa* cell attachment onto the surface. As shown in Figure 7 and Table 7, surfaces containing 5% additives were found to be most biocidal, this may due to the membrane stretching due to the differential gradient of surface tensions at nanoscale, leading to the instability of membrane integrity [21,22,38]. Similar effects were observed for surfaces with 5% additives against *S. aureus*. The synergetic combination between surface topography and differential gradient of surface tensions leads to the maximized effect of antifouling and biocidal effects. 

As discussed previously, the biocidal and antimicrobial activity of Control 1 coating, e.g., in the absence of aluminum silicate surface-treated titanium dioxide microparticles, did not increase through the siloxane modification process. This may be due to several reasons. For example, the titanium dioxide provides additional roughness (micro and nanostructure) to the surface of the cured coating, which imparts some bactericidal activity to the surface. There may also be some synergistic effects taking place between the pigment and the polymeric agglomerates formed as a result of the presence of the siloxane additive. The pigmentation may also provide some crowding effects that drive self-assembly and migration of siloxane to the surface, thereby enhancing its bactericidal behavior. 

The presence of siloxane (co)polymers in coatings has been commonly accepted as a contributing factor towards reducing the extent of cell attachment [39], however, in this study, a significant increase in the biocidal activity against both *P. aeruginosa* and *S. aureus* bacteria was observed for the pigmented coatings that contained a 5 wt.% additive siloxane. This result was unexpected, given that no inorganic/organic biocides were incorporated in the coating composition formulation. To further investigate the reasons behind the increased biocidal activity of this coating, additional studies of the surface topography were undertaken in both air and liquid, the results of which are discussed in the following sections.

### 3.3. Dynamic Topographical Characteristics of the Modified Coating

To investigate the unexpected biocidal activity of the coatings containing the 5 wt.% siloxane additive, the nano-topography and nano-mechanical properties of the coating were studied in both air and a buffered aqueous solution using in the PeakForce QNM. The buffered solution was comprised of 50 mM Tris-HCl, 50 mM glycine and 200 mM KCl.

**Figure 8:**
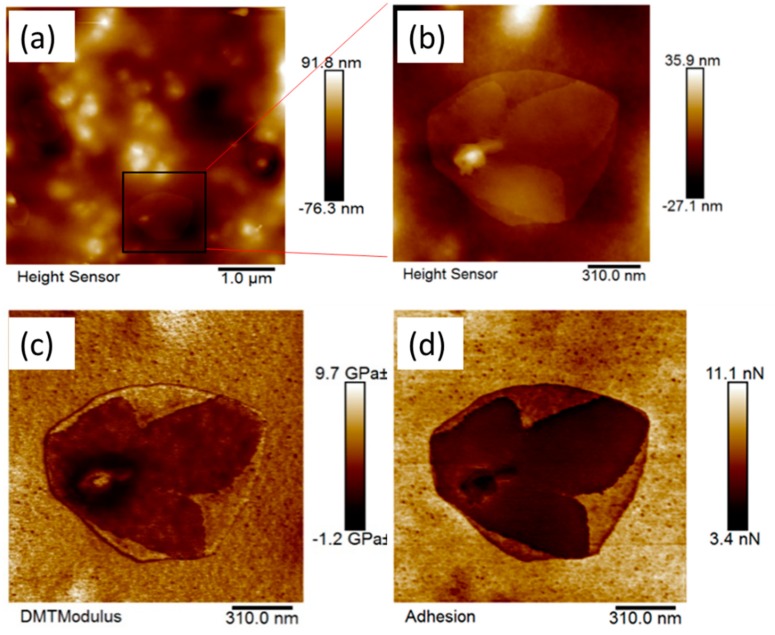
AFM images of the Control 2 coating with 5 wt.% siloxane additives in the air (**a**), AFM images (1.5 µm image size) showing the plate-like surface features (**b**) height (**c**) modulus and (**d**) adhesion map.

The AFM height images of the modified coating in the air (Figure 8) show the presence of titanium dioxide particles at the surface, in addition to a number of other micro- and nanofeatures. Thin plate-like (discoid) features in the 5 µm range can be seen in Figure 8a. These nano-features were not detected on the surface of the Control 2 coating (pigmented coating composition with no modification) as shown in Figure S3. The stiffness (modulus) and adhesion images shown in Figure 8c,d reveal the presence of sub-nanostructure features inside these plate-like (discoid) features. Interestingly, these plate-like nanofeatures exhibited a lower modulus and adhesion than that of the features in the surrounding areas.

**Figure 9:**
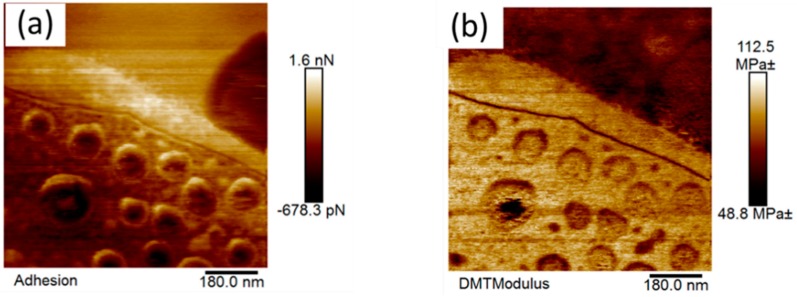
(**a**) AFM adhesion force mapping and (**b**) modulus mapping of the Control 2 coating with the 5 wt.% additive analyzed in water.

The 5 µm size AFM images obtained for the coating immersed in liquid highlighted some smaller discoid-like nanofeatures, in addition to the plate-like nanofeatures that appeared in the images taken of the coating in air. Interestingly, these new nanofeatures possessed different micromechanical properties (Figure 9a). The emergence of these new nanofeatures in the liquid environment suggested the coatings possessed a stimuli-responsive nature, an attribute that may have contributed to the biocidal activity of the coating.

As described above, mapping of the elastic modulus for this coating revealed a mechanical contrast between the polyester-melamine resin, siloxane domains, and areas of the surface containing the titanium dioxide particles. A comparison of the results obtained in water and air revealed that the modulus was lower in the water environment. The modulus varied from very small values up to a maximum of approximately 10 GPa for the 1.5 µm sized image (Figure 8c) taken in air, whereas for the coating immersed in water, the maximum elastic surface modulus was found to be approximately ten-fold lower, around 0.11 GPa, as presented in Figure 9b.

The greatest measured modulus in the water environment was found to be comparable to the elastic modulus of PDMS [40], whereas the measured moduli in the presence of air were comparable to those of stiff polymers such as polyester and polystyrene [41]. A similar trend was observed for the adhesion force, which was approximately 10 times lower in the water environment (~0.6–1 nN) compared to that obtained in the air (~5–9 nN) (see the adhesion map profile, Figure 8d). 

The size and spacing between the discoid-like nano-features were found to also be dependent on the phase in contact with the coating. The results presented in Table 8 show that both the size and spacing between the nanofeatures when in contact with water, were smaller than those observed when the coating was in contact with air. This observation shows the interesting dynamic nano-structural nature of coatings made by blending siloxane additives (TEGOMER^®^ MSi 2650) in thermoset polyester-melamine polymers. 

**Figure 10:**
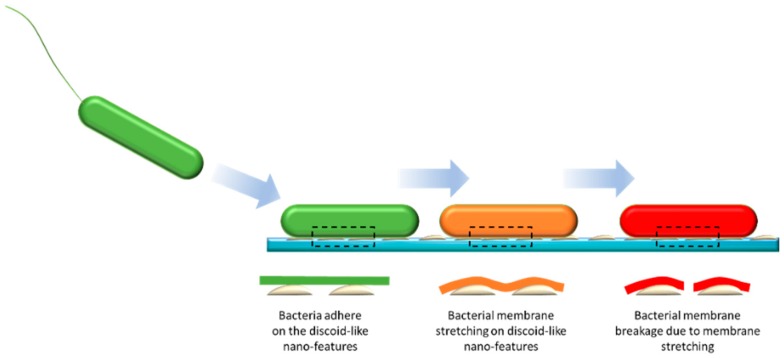
Hypothesized antibacterial mechanisms of discoid-like nanofeatures: bacteria adhere to discoid-like nano-features, followed by bacterial membrane stretching leading to the breakage of bacterial membranes.

It is postulated that the homogenous modified thermoset polyester solution, which underwent polymerization-induced phase segregation and self-assembly on the surface, formed a different nanostructure when placed in contact with water (Figure 10). Consequently, rather than imparting the preventative adhesion properties often observed for continuous polysiloxane surfaces [42,43], these nano-topologies resulted in a significant degree of bactericidal properties to the surface. Given that a modified coating based on non-pigmented coating composition (“Control 1”) was inactive against both strains of bacteria, we conclude that the presence of the titanium dioxide particles in the pigmented coating caused the unexpected biocidal effect. Considering that the titanium dioxide microparticles used as pigment in the coating composition was surface treated with aluminum silicate (to suppress any photoactivity [13]) and that antimicrobial testing was performed in a dark incubator, this effect supported our assertion that dual-scale roughness, e.g. micro- and nano-roughness was important in achieving the anti-microbial effect. We have implemented detailed studies of the micromechanical analysis of these nanofeatures to explore their bactericidal activities, and this work will be presented elsewhere.

## 4. Conclusions

In this study, the incorporation of a siloxane copolymer additive poly((2-phenylethyl) methylsiloxane)-co(1-phenylethyl) methylsiloxane)-co-dimethylsiloxane) into non-pigmented and pigmented polyester-melamine resin was investigated. 

We demonstrated that the incorporation of the siloxane copolymer into the pigmented coating composition in the range of 5–11 wt.% relative to the solids content of the coating composition altered the surface morphology significantly relative to a similar non-pigmented coating and improved the self-cleaning of the coating compared to control coatings without additive. 

An XPS analysis of the coatings highlighted that siloxane migrated to the surface after a rapid curing process as a result of polymerization induced phase segregation. Increasing the concentration of the siloxane additive resulted in the formation of a surface with a higher concentration of siloxane. For a coating with 11 wt.% siloxane additive, the XPS peak intensity for nitrogen (which arises from the polyester-melamine resin) was found to have diminished compared to the coating without the additive, indicating that siloxane comprised the top 5–10 nm of the surface. Consequently, the surface of the coating became more hydrophobic, had lower surface energy and was less polar. Topographical images showed that the modified polyester-siloxane coating at the sub-micron level had a rougher surface than that of the control polyester resin. 

The unexpected finding, however, was the biocidal activity exhibited by the modified pigmented coating. This activity of the coating arose in the absence of any post-treatment and/or incorporation of biocidal chemicals, displaying biocidal activity against *Pseudomonas aeruginosa* and *Staphylococcus aureus* bacteria. For coatings prepared with 5 wt.% siloxane, approximately 65% of all the cells attaching to the surface were inactivated, compared to approximately 8 and 15% (respectively) on the control polymer resin. Investigation of the topographical and micromechanical characteristics of the siloxane-containing coatings in both air and water by AFM highlighted the presence of discoid nano-features and revealed the stimuli-responsive nature of these nano-features.

In summary, the pigmented polyester-siloxane copolymer coatings prepared by a simple one-pot fabrication method exhibited the specific nano-roughness and morphological attributes that confer with good self-cleaning and, in one case, excellent biocidal activity. The biocidal performance was achieved without the addition of any toxic biocidal additives or post-treatment methods (to introduce the nano-textures) commonly used to fabricate antimicrobial coatings. In many applications, incorporation of biocidal compounds is not allowed, and hence, this simple method of fabricating an environmentally friendly biocidal coating would be potentially very attractive to many industries and organizations. This fabrication approach provides the opportunity to create a coating that can control the nano-topography of the surface through the incorporation of suitable siloxane copolymers for different types of bacteria and applications. 

## Figures and Tables

**Table 1:** Properties of coatings based on non-pigmented coating formulation (Control 1) without and with 5% of the siloxane additive.

Coating Composition Code	Segregating Additive (wt. %)	Water Contact Angle (°)	Glycerol Contact Angle (°)	Diiodomethane Contact Angle (°)	Surface Energy (mN/m)
Control 1	-	80.2 ± 4.8	79.4 ± 2.0	56.4 ± 2.0	32.1
With siloxane additive	5	93.1 ± 0.9	85.1 ± 1.8	40.2 ± 1.5	42.2

**Table 2:** Antibacterial performance of coated surfaces based on the Control 1 formulation.

Sample Code	Percentage of Siloxane Additive (wt.%)	*Pseudomonas aeruginosa*		*Staphylococcus aureus*	
		Non-Viable Cells, %	Cell Density (Cell Number × 10^4^)	Non-Viable Cells, %	Cell Density (Cell Number × 10^4^)
**Control 1**	-	50	18	42	0.5
with siloxane additive	5	22	20	40	2.6

**Table 3:** Surface properties for Control 2 with and without incorporation of the siloxane additive.

Segregating Additive (wt.%)	Water Contact Angle (°)	Glycerol Contact Angle (°)	Diiodomethane Contact Angle (°)	Surface Energy [mN/m]
0 (Control 2)	80.2 ± 1.4	80.1 ± 1.5	29.7 ± 1.3	59
5	90.6 ± 1.3	88.8 ± 2.4	37.2 ± 2.8	48
9	105.3 ± 2.8	94.2 ± 4.4	57.4 ± 2.5	31
11	103.4 ± 1.4	96.0 ± 3.2	55.8 ± 2.1	33

**Table 4:** Surface chemistry (at%) of coating compositions containing different concentrations of TEGOMER^®^ M-Si 2650 (Control 1 and Control 2).

Coating Composition	Si (Organic)	N	C	O
Pigmented coating composition (Control 2)	0.2	1.3	78.1	20.4
Control 2 with 5 wt.% additive	9.7	0.8	68.6	20.9
Control 2 with 11 wt.% additive	19.4	<dl ^a^	60.9	19.7
Control l with 5 wt.% additive	6.5	4.4	67.4	21.7

^a^ <dl: below detection limit.

**Table 5:** Dirt pick-up (DPU) results for Control 2 formulations without and with siloxane additive.

Siloxane Additive, wt.%	DPU (Delta L)
0 (Control 2)	−30.1
5	−17.9
9	−13.4
11	−16.4

**Table 6:** Surface roughness parameters (in nm) for the Control 2 coating and the corresponding modified coatings in air for an atomic force microscope (AFM) analysis area of 1 µm^2^.

Copolymer Siloxane Additive (wt.%)	R_rms_	R_a_	R_max_
0	4.53 ± 3.6	3.4 ± 2.6	39.5 ± 23.7
5	13.6 ± 1.6	11.0 ± 1.3	72.4 ± 5.5
11	14.0 ± 4.2	11.0 ± 3.0	118.0 ± 45.0

**Table 7:** Antimicrobial performance of modified coatings based on the Control 2 (pigmented coatings) with and without siloxane additives.

Additive (wt.%)	*P. aeruginosa*		*S. aureus*	
	Non-Viable Cells, %	Cell Density (Cell Number × 10^4^)	Non-Viable Cells, %	Cell Density (Cell Number × 10^4^)
0	15	12.7	7.7	2.3
5	64	5.6	66	7.1
11	4.9	0.8	9	4.4

**Table 8:** Nanofeature specifications for the Control 2 coating with 5 wt.% siloxane additives in air and water.

Sample Environment	Feature Shape	Size (nm)	Height Thickness (nm)	Spacing Distance (nm)
in air	discoid	500–1200	5–15	1000–2000
in water	discoid	80–180	1–3	40–150

## Supplementary Materials

The following are available online at https://www.mdpi.com/2079-4991/9/11/1610/s1, Figure S1: FESEM image of titanium dioxide particles present in Control 2 sample modified with 5 wt.% siloxane additives. Figure S2: Illustration of “Control 1” and “Control 2” coatings. Figure S3: AFM image of the Control 2 coating (unmodified pigmented coating); Table S1: Titanium dioxide particles size measured by ImageJ from Figure S1; Table S2: The surface tensions and their components for the wetting liquids at 20 °C; Table S3: Ratings for the dirt resistance test.
