# Supplementary material for: Polymerization-Induced Phase Segregation and Self-Assembly of Siloxane Additives to Provide Thermoset Coatings with a Defined Surface Topology and Biocidal and Self-Cleaning Properties
Source: Nanomaterials (Basel). 2019 Nov 13;9(11):1610. doi: 10.3390/nano9111610 (PMC6915580; doi:10.3390/nano9111610)
Supplement: Supplementary file 1 [file nanomaterials-09-01610-s001.docx]

# Supporting Information

Polymerization-Induced Phase Segregation and Self-Assembly of Siloxane Additives to Provide Thermoset Coatings with a Defined Surface Topology and Biocidal and Self-Cleaning Properties

Jaleh Mansouri ^1,2,^*, Vi Khanh Truong ^2,3,4^, Shane MacLaughlin ^5^, David E. Mainwaring ^3^, Graeme Moad ^6,^*, Ian J. Dagley ^2,7^, Elena P. Ivanova ^3,4^, Russell J. Crawford ^4^ and Vicki Chen ^1,8^

^1^ UNESCO Centre for Membrane Science and Technology, School of Chemical Engineering, University of New South Wales, Sydney, NSW 2052, Australia

^2^ Cooperative Research Centre for Polymers, Notting Hill, VIC 3168, Australia; [vi.khanh.truong@rmit.edu.au](mailto:vi.khanh.truong@rmit.edu.au) (V.K.T.); [dagley@bigpond.net.au](mailto:dagley@bigpond.net.au) (I.J.D.)

^3^ Faculty of Science, Engineering and Technology, Swinburne University of Technology, PO Box 218, Hawthorn, VIC 3122, Australia; [demainwaring@swin.edu.au](mailto:demainwaring@swin.edu.au) (D.E.M.); [elena.ivanova@rmit.edu.au](mailto:elena.ivanova@rmit.edu.au) (E.P.I.)

^4^ Nanobiotechnology Laboratory, School of Science, College of Science, Engineering and Health, RMIT University, Melbourne, VIC 3001, Australia; [russell.crawford@rmit.edu.au](mailto:russell.crawford@rmit.edu.au) (R.J.C.)

^5^ BlueScope Steel Research, Port Kembla, NSW 2505, Australia; [Shane.Maclaughlin@bluescopesteel.com](mailto:Shane.Maclaughlin@bluescopesteel.com) (S.M.)

^6^ CSIRO Manufacturing, Clayton, VIC 3168, Australia; [graeme.moad@csiro.au](mailto:graeme.moad@csiro.au) (G.M.)

^7^ Defence Science and Technology, Department of Defence, 506 Lorimer Street, Port Melbourne, VIC 3207, Australia

^8^ School of Chemical Engineering, University of Queensland, Brisbane, QLD 4072, Australia; [v.chen@uq.edu.au](mailto:v.chen@uq.edu.au) (V.C.)

***** Correspondence: j.mansouri@unsw.edu.au; [graeme.moad@csiro.au](mailto:graeme.moad@csiro.au) (G.M.); Tel.: +61-2-93854328 (J.M.); Tel +61-3-95452509 (G.M.)

-
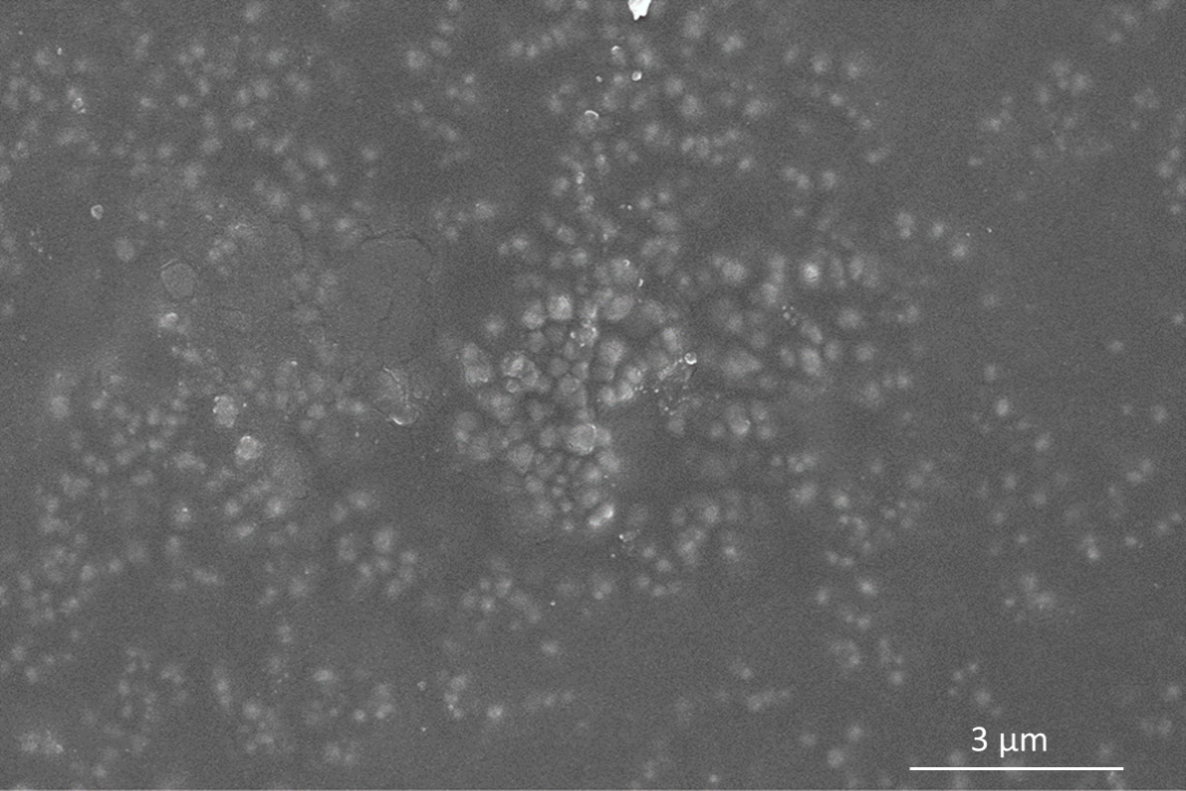


**Figure S1.** FESEM image of titanium dioxide particles present in Control 2 sample modified with 5 wt.% siloxane additive.

Table S1. Titanium dioxide particles size measured by ImageJ from Figure S1

| Magnification | 25k |
| --- | --- |
| 1 | 0.244 |
| 2 | 0.315 |
| 3 | 0.265 |
| 4 | 0.316 |
| 5 | 0.304 |
| 6 | 0.213 |
| 7 | 0.366 |
| 8 | 0.283 |
| 9 | 0.203 |
| 10 | 0.303 |
| 11 | 0.244 |
| 12 | 0.206 |
| Mean | 0.272 |
| SD | 0.049 |

Analysed by ImageJ software.

**SI-1: Detailed modified coating composition preparation method**

1. A required amount of siloxane copolymer was added to 2-butanol and probe sonicated for 5 minutes at 20% amplitude. The vial was placed in ice-water bath to minimise heating of the solution.

2. Polyester-melamine coating solution (Control 1 and Control 2) was stirred with a mechanical stirrer and added to the siloxane copolymer solution in step 1 above to form the modified coating solution.

3. The modified coating solution was then probe sonicated based on the procedure given below:

Conditions of probe sonication: Amplitude: 30%, 40 seconds pulse ON/ 7 seconds pulse OFF for 20 minutes. The coating composition sample vial was kept in ice-water bath during the duration of the probe sonication to avoid heating the solution.


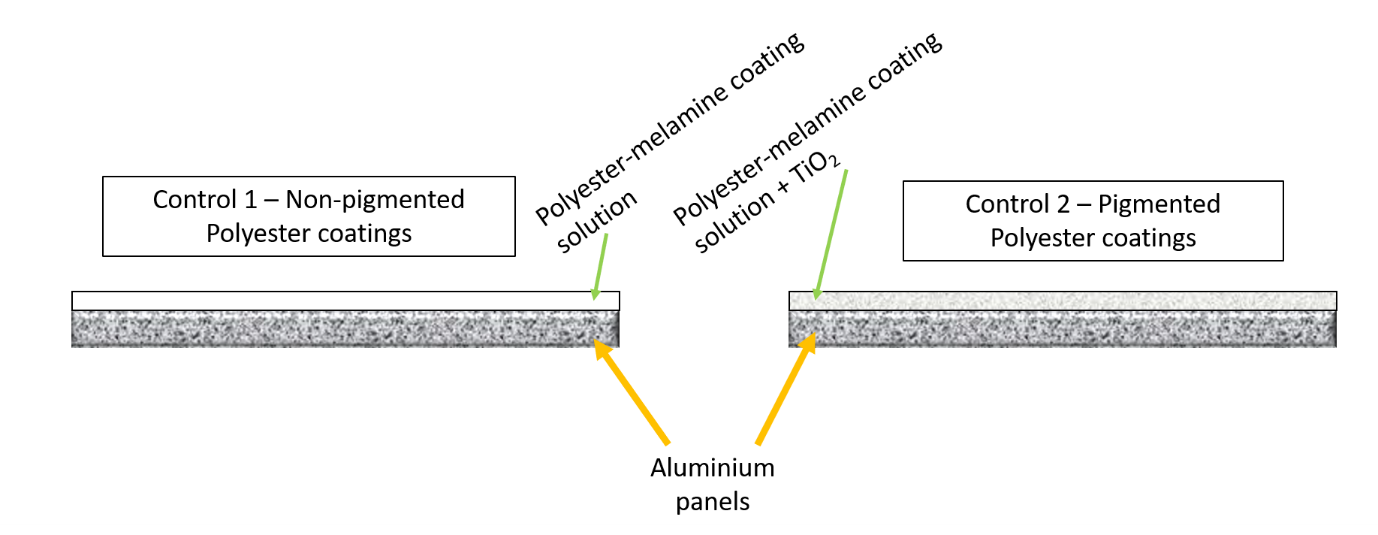


**Figure S2.** Illustration of “Control 1” and “Control 2” coatings.

***SI-2 Surface energy calculation***

The surface energy of the coatings was determined using *Van Oss-Chaudhury-Good* theory and Equation 1.

$0.5\gamma_{L}\left( 1+cos\theta\right)=\left( \gamma_{S}^{D}\gamma_{L}^{D} \right)^{1/2}+\left( \gamma_{S}^{-}\gamma_{L}^{+} \right)^{1/2}+\left( \gamma_{S}^{+}\gamma_{L}^{-} \right)^{1/2}$ (1)

where $\gamma$ refers to surface energy, $\theta$ is the contact angle, the subscripts S and L refer to solid and wetting liquid, and the superscripts D, + and – refer to dispersive, acid and base components.

To determine the three unknowns in the equation, namely$\gamma_{S}^{D}$,$\gamma_{S}^{-}$ and $\gamma_{S}^{+}$, three types of wetting liquids are required. In this work, methylene iodide, water, and glycerol were used and the values for their surface energy components are shown in the table below.

Table S2. The surface tensions and their components for the wetting liquids at 20°C.

| **Liquid** | $\boldsymbol{\gamma}_{\boldsymbol{L}}$ **(mN/m)** | $\boldsymbol{\gamma}_{\boldsymbol{L}}^{\boldsymbol{D}}$**(mN/m)** | $\boldsymbol{\gamma}_{\boldsymbol{L}}^{\boldsymbol{+}}$**(mN/m)** | $\boldsymbol{\gamma}_{\boldsymbol{L}}^{\boldsymbol{-}}$**(mN/m)** |
| --- | --- | --- | --- | --- |
| Methylene iodide | 50.8 | 50.8 | 0 | 0 |
| Glycerol | 64.0 | 34.0 | 3.92 | 57.4 |
| Water | 72.8 | 21.8 | 51.0 | 25.5 |

**SI-3: Detailed dirt resistance test method**

A slurry of activated carbon and water was prepared by mixing 15 part of carbon powder in 85 parts of water by weight. The slurry was applied to the coated surface (with a small spatula) to cover a roughly 20-30 mm diameter area. Samples were then placed in a 70°C oven for one hour to dry the slurry. The excess carbon was removed by shaking the sample plate from edges (tapping at the back of plates 5 times). Then sample were washed under running water for 6 times, following with washing and light brushing for 10 times. The dirt resistance was quantified using dirt pick-up (DPU) analysis (also known as carbon slurry test). CIE (∆L), which is the difference in colour of the paint sample before and after contamination, measured by calorimeter, quantifies dirt resistance according to the scale below.

Table S3. Ratings for the dirt resistance test.

| **Rating** | **CIE* (∆L)** |
| --- | --- |
| Excellent | 0 to -1 |
| Very Good | < -1 to -5 |
| Good | < -5 to -10 |
| Moderate | < -10 to -20 |
| Fair | < -20 to -30 |
| Poor | < -30 |

* CIE refers to the Commission internationale de l'éclairage (CIE) which is the International Commission on Illumination. This organization is responsible for the international coordination of lighting related technical standards.

**SI-4 Antimicrobial analysis**

*Bacterial growth conditions and sample preparation*

Two strains were used: *Pseudomonas aeruginosa* (PA) ATCC 9721 and *Staphylococcus aureus* (SA) CIP 65.8^T^. The selected strains are typical representatives of two large bacterial taxonomic lineages and were obtained from American Type Culture Collection (ATCC, Manassas, VA, USA) and Culture Collection of the Institute Pasteur (CIP, Paris, France) respectively. Prior to each experiment, bacterial cultures were refreshed on nutrient agar from stocks (Oxoid, Basingstoke, Hampshire, UK). Fresh bacterial suspensions were grown overnight at 37 °C in 5 mL of nutrient broth (Oxoid, Basingstoke, Hampshire, UK). Fresh bacterial suspensions were prepared prior to each surface analysis from which cells were collected at the logarithmic stage of growth and adjusted to OD600 = 0.1 prior to each experiment, as detailed elsewhere (^26^). The surfaces (5 mm × 5 mm) will be placed in 24 well plates (Thermo Scientific), and 2 mL of bacterial suspensions were added into the well. The whole 24well plates were incubated in dark incubator at 25 °C.

*Bacterial growth and surface bacterial assays*

Cell attachment densities and viability were assessed by confocal laser scanning microscopy (Fluoview FV 10i, Olympus, Japan) using a mixture of SYTO 9 and propidium dyes in a LIVE/DEAD BacLight Bacterial Viability Kit, L7012, where SYTO 9 permeated both intact and damaged cell membranes fluorescing green, but propidium iodide only entered cells with sufficiently damaged membranes fluorescing red indicating non-viability.


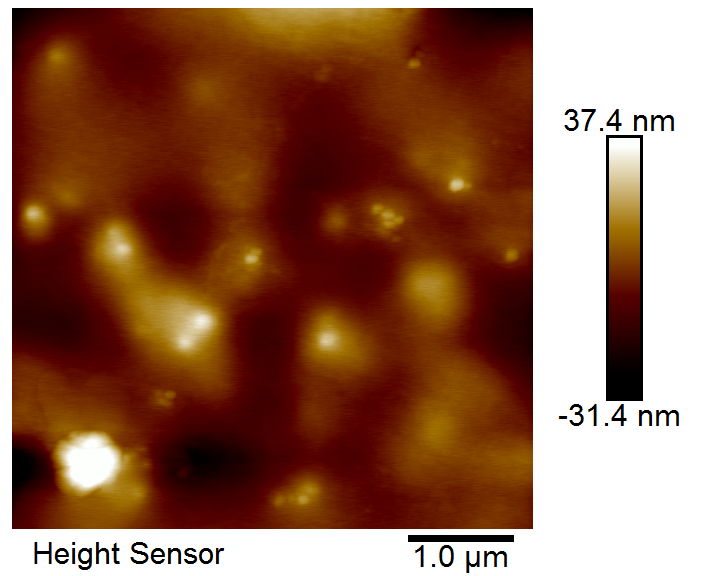


**Figure S3.** AFM image of the Control 2 coating (unmodified pigmented coating)
